# MGMT methylation may benefit overall survival in patients with moderately vascularized glioblastomas

**DOI:** 10.1007/s00330-020-07297-4

**Published:** 2020-10-01

**Authors:** Elies Fuster-Garcia, David Lorente Estellés, María del Mar Álvarez-Torres, Javier Juan-Albarracín, Eduard Chelebian, Alex Rovira, Cristina Auger Acosta, Jose Pineda, Laura Oleaga, Enrique Mollá-Olmos, Silvano Filice, Paulina Due-Tønnessen, Torstein R. Meling, Kyrre E. Emblem, Juan M. García-Gómez

**Affiliations:** 1 Department of Diagnostic Physics, Oslo University Hospital, Sognsvannsveien 20, 0372 Oslo, Norway; 2 Medical Oncology Service, Hospital Provinicial de Castellón, Castellón de La Plana, Castellón, Spain; 3 Instituto Universitario de Tecnologías de la Información y Comunicaciones, Universitat Politècnica de València, València, Spain; 4 Section of Neuroradiology, Hospital Universitari Vall d’Hebron, Barcelona, Spain; 5 Hospital Clínic, Barcelona, Spain; 6 Hospital Universitario de La Ribera, València, Spain; 7 Department of Medical Physics, University Hospital of Parma, Parma, Italy; 8 Department of Radiology, Oslo University Hospital, Oslo, Norway; 9 Department of Neurosurgery, Oslo University Hospital, Oslo, Norway; 10 Department of Neurosurgery, Geneva University Hospitals, Geneva, Switzerland

**Keywords:** Perfusion imaging, Glioblastoma, *O(6)-Methylguanine-DNA methyltransferase*, Prognostic factors, Temozolomide

## Abstract

**Objectives:**

To assess the combined role of tumor vascularity, estimated from perfusion MRI, and *MGMT* methylation status on overall survival (OS) in patients with glioblastoma.

**Methods:**

A multicentric international dataset including 96 patients from NCT03439332 clinical study were used to study the prognostic relationships between *MGMT* and perfusion markers. Relative cerebral blood volume (rCBV) in the most vascularized tumor regions was automatically obtained from preoperative MRIs using ONCOhabitats online analysis service. Cox survival regression models and stratification strategies were conducted to define a subpopulation that is particularly favored by *MGMT* methylation in terms of OS.

**Results:**

rCBV distributions did not differ significantly (*p* > 0.05) in the methylated and the non-methylated subpopulations. In patients with moderately vascularized tumors (rCBV < 10.73), *MGMT* methylation was a positive predictive factor for OS (HR = 2.73, *p* = 0.003, AUC = 0.70). In patients with highly vascularized tumors (rCBV > 10.73), however, there was no significant effect of *MGMT* methylation (HR = 1.72, *p* = 0.10, AUC = 0.56).

**Conclusions:**

Our results indicate the existence of complementary prognostic information provided by *MGMT* methylation and rCBV. Perfusion markers could identify a subpopulation of patients who will benefit the most from *MGMT* methylation. Not considering this information may lead to bias in the interpretation of clinical studies.

**Key Points:**

*• MRI perfusion provides complementary prognostic information to MGMT methylation.*

*• MGMT methylation improves prognosis in glioblastoma patients with moderate vascular profile.*

*• Failure to consider these relations may lead to bias in the interpretation of clinical studies.*

**Electronic supplementary material:**

The online version of this article (10.1007/s00330-020-07297-4) contains supplementary material, which is available to authorized users.

## Introduction

Gliomas are the most common malignant primary central nervous system tumors, with an estimated annual incidence of 3.21 per 100,000 individuals in the USA [1]. About half of all newly diagnosed gliomas are classified as glioblastoma, which is the most malignant type of brain cancer. Glioblastoma pathology is characterized by angiogenesis, highly infiltrative growth, and cellular heterogeneity [2]. Despite an aggressive therapeutic approach combining maximum safe resection with radiotherapy (RT) plus concomitant and adjuvant temozolomide (TMZ), prognosis is poor, with median overall survival (OS) duration of approximately 14 months [3]. In 2016, the World Health Organization introduced molecular parameters along with histology to describe the interpatient glioblastoma heterogeneity associated with differential prognosis and responses to therapy [4].

Adequate clinical and molecular biomarkers are needed for accurate estimations of prognosis and optimal treatment selections. Inactivation through promoter methylation of the O^6^-methylguanine-DNA methyltransferase (*MGMT*) gene, which impairs the ability to repair DNA damaged induced by alkylating agents such as TMZ [5], has been described as a relevant biomarker for clinical decision-making in glioblastoma treatment. In a post hoc analysis of a phase III trial, *MGMT* promoter methylation was associated with a 2-year survival increase in TMZ-treated glioblastoma patients from 14 to 46% [6]. Additional studies have also described not only a predictive, but also a prognostic role of *MGMT* methylation for glioblastoma patients [7, 8]. Current guidelines support the use of *MGMT* methylation as a predictive biomarker in patients older than 70 years with isocitrate dehydrogenase (IDH) wild-type grade IV gliomas [8].

Despite the well-documented impact of *MGMT* methylation on the prognosis of glioblastoma patients treated with TMZ, the survival of these patients is not explained by this factor alone. Tumor vascularity, for instance, is strongly associated with glioma transformation (i.e. grade progression), poorer survival [9], sensitivity to RT, and effectiveness of bloodborne delivery of nutrients and chemotherapy [10].

MRI perfusion–based parameters correlate with tumor vascularity and properties of vessels [11, 12]. Numerous studies show that the vascularity as defined by MRI perfusion is a prognostic factor for glioblastoma even prior to initial surgery [13–15]. Perfusion parameters, such as relative cerebral blood volume (rCBV) in the enhancing tumor areas, are among the most consistently recognized independent predictors of survival [9]. Novel approaches based on artificial intelligence [15, 16] have been proposed to calculate perfusion-based biomarkers, based not on the entire enhancing lesion but on more homogeneous regions (habitats), thereby improving not only the prognostic capacity but also the reproducibility of the results [13, 17, 18].

In this study, we aimed at evaluating whether the rCBV is a modulating factor of the prognostic effect of MGMT methylation status in patients with glioblastoma treated with TMZ.

## Materials and methods

### Patient cohort

**Fig. 1:**
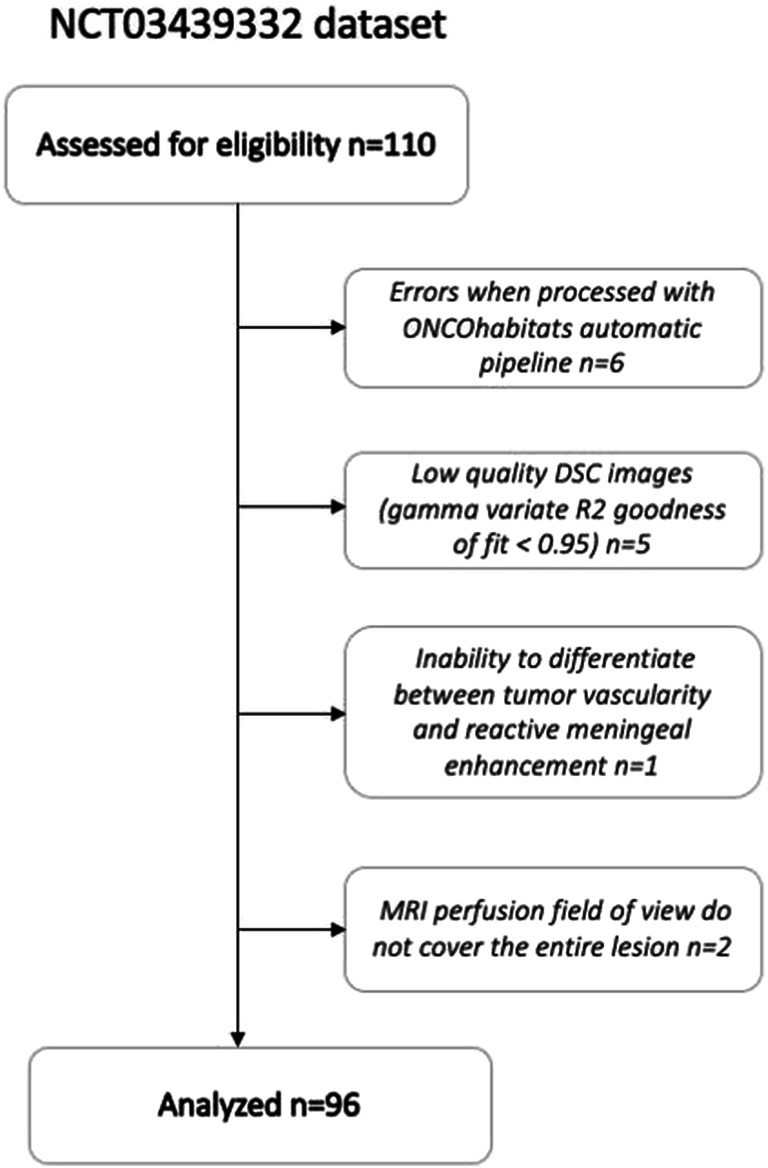
CONSORT diagrams for the NTC3439332 dataset

The patient cohort has compiled by the multicenter and international retrospective clinical study NCT03439332 [19]. A total of 110 cases from this dataset with MRI perfusion studies were included in the current study (Fig. 1). The NCT03439332 multicenter international dataset included patients treated at seven European hospitals from four countries: Hospital Clinic, Barcelona, Spain; Hospital Universitario Vall d’Hebron, Barcelona, Spain; Hospital Universitario de La Ribera, Alzira, Spain; Hospital de Manises, Manises, Spain; Azienda Ospedaliero-Universitaria di Parma, Parma, Italy; Oslo University Hospital, Oslo, Norway; and Centre Hospitalier Universitaire de Liège, Liège, Belgium. Patients were diagnosed with glioblastoma grade IV WHO with histopathological confirmation and followed Stupp standard treatment. The extent of resection was assessed in each center by expert neurosurgeons and radiologists based on the postsurgical MRI study findings. A material transfer agreement was approved by all the participating centers and an acceptance report was issued by the Ethical Committee of each center. The Universitat Politècnica de València institutional ethical board also approved this retrospective study. All methods were performed in accordance with the relevant guidelines and regulations or Declaration of Helsinki.

### *IDH1* mutation and *MGMT* methylation status assessment

*MGMT* methylation status was assessed in each center by pyrosequencing using a cut-off value between 9 and 10%, except for the cases from the Hospital Clinic in Barcelona where *MGMT* methylation status was assessed by methylation-specific polymerase chain reaction. The *IDH1* mutation status was assessed in each center by immunostains using the *IDH1* R132H antibody.

### MRI data acquisition protocol

Pre-surgical standard-of-care MRI examinations including pre- and post-gadolinium T1-weighted MRI as well as T2-weighted, T2-fluid-attenuated inversion recovery (FLAIR), and dynamic susceptibility contrast (DSC) T2* perfusion-weighted sequences were collected from each participating center. All the MRIs were obtained by 1.5-T or 3-T scanners, using different MRI acquisition protocols in each participating center. A summary of the MRI acquisition protocol used by each center is presented in Supplementary Materials Table S-I.

### MRI data preprocessing

Anatomical MRI acquisitions were preprocessed using the following pipeline: (1) voxel isotropic resampling, (2) denoising, (3) rigid intra-patient registration, (4) affine registration to MNI space [20], (5) brain extraction, and (6) magnetic field inhomogeneity correction. First, image resampling at 1 mm^3^ was performed through linear interpolation. Next, denoising was carried out using the adaptive non-local means filter [21] with a search window of 7 × 7 × 7 voxels and a patch window of 3 × 3 × 3 voxels. Registration was performed with ANTs software [22] and Mutual Information, using the T1c MRI as reference. Brain extraction was performed through a convolutional neural network with a U-Net architecture trained on a dataset of 160 T1c MRIs with glioblastomas manually annotated and validated by several independent neuroimaging researchers with more than 5 years of experience. Finally, magnetic field inhomogeneities were corrected with the N4 software using the previously computed intra-cranial mask [23].

### Lesion segmentation

Glioblastoma tissue segmentation was performed by means of 3D patch-based convolutional neural networks [24]. A U-Net Res-Net architecture of 5 levels with long-term skip connections with 16, 32, 64, 128, and 256 filters at each level, respectively, was used to perform the segmentation. At each level, a simple block and a residual block were chained together. A simple block consists of a convolution + batch normalization + ReLu activation function. A residual block consists of a convolution + batch normalization + ReLu + convolution + batch normalization + residual connection summation + ReLu activation function. Convolutions were performed with isotropic kernels of size 3 × 3 × 3, while batch normalization momentum was fixed to 0.9. Max-pooling layers with pooling size and stride 2 × 2 × 2 were employed in the contracting path to sequentially condensate the relevant features of the input patches, while transpose convolutions were used in the expanding path to rearrange and project the latent features to the original size. The network works with patches of 32 × 32 × 32 with 3 channels corresponding to the T1c, T2, and FLAIR sequences. Adam optimizer with cross-entropy loss-function was used to train the network. The lesion segmentation network was trained on BraTS dataset [25–27] including 260 MRI studies segmented manually, by one to four raters, following the same annotation protocol, and their annotations were approved by experienced neuroradiologists. Based on a blind independent validation dataset provided by BraTS international challenge, the results of this segmentation method obtain a median DICE of 0.85 on delineating the enhancing tumor region and a DICE of 0.92 in delineating the whole tumor region [28].

### DSC perfusion quantification

Quantification of rCBV and relative cerebral blood flow (rCBF) hemodynamic indices was performed employing standard techniques proposed in the literature [29]. rCBV was calculated by numerical integration of the area under the curve of the T2* concentration-time signal, while rCBF was obtained by means of the block-circulant singular value decomposition (SVD) devolution technique [29]. Gamma-variate curve-fitting and Boxerman technique [30] were used to correct for T2 and T1 leakage effects. The mean transit time was computed from the central limit theorem by dividing rCBV/rCBF. The arterial input function (AIF) was automatically detected by means of an iterative divide-and-conquer approach, using the peak height, the time-to-peak, and the full-width at half maximum as features to detect arterial shape-like signals [24].

### Definition of the vascular marker

**Fig. 2:**
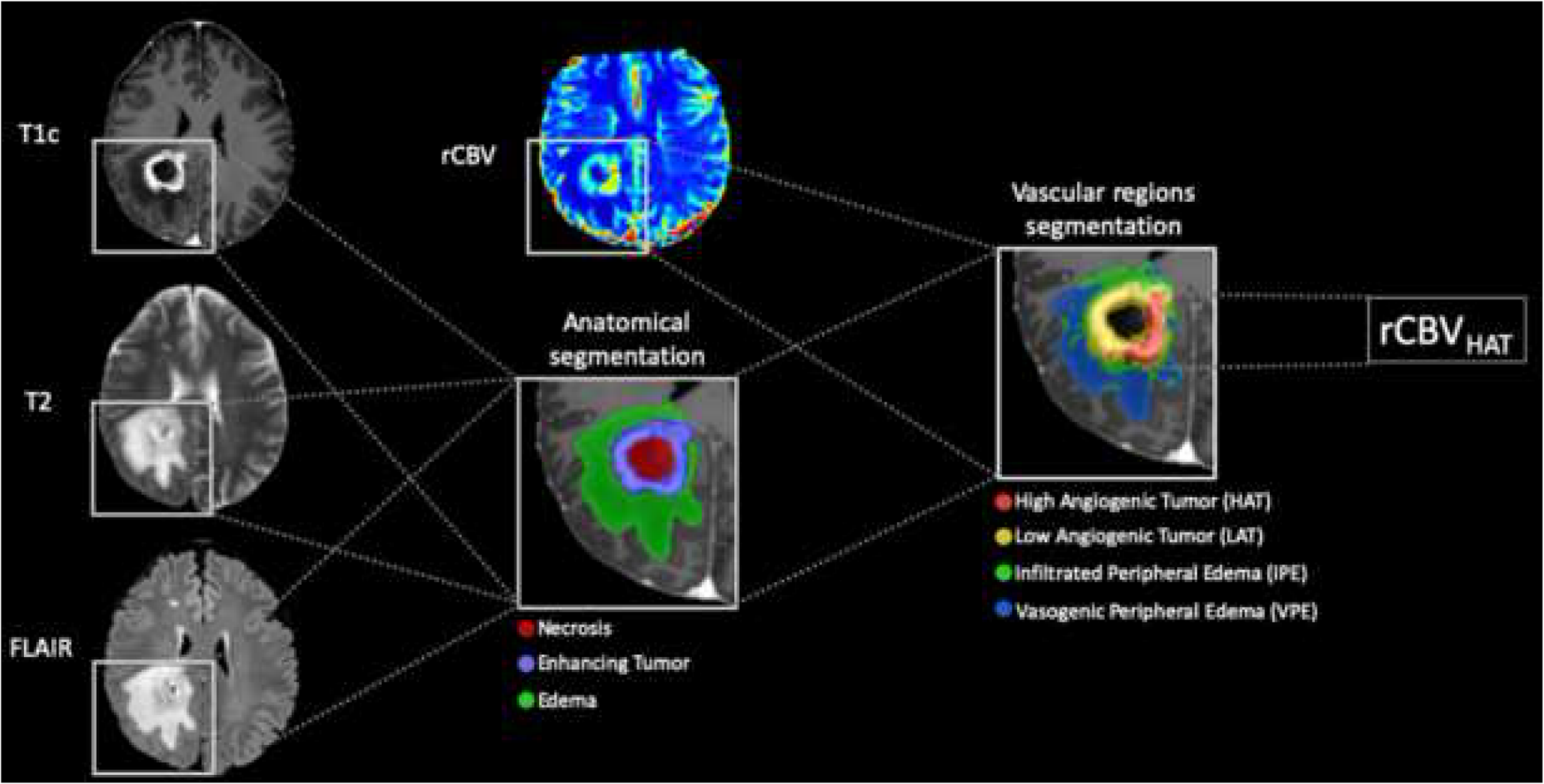
Schema of the methodology used to compute the relative cerebral blood volume at the high angiogenic tumor (rCBV_HAT_) region

The vascular marker used was based on the rCBV map obtained from the DSC MRI sequence. Specifically, we used the 90th percentile of rCBV values at the highly angiogenic tumor (HAT) region of the tumor (rCBV_HAT_) as a robust indicator of the maximum perfusion value of the region. The HAT region was defined based on rCBV and rCBF maps following the methodology proposed by Juan-Albarracín et al [15] and implemented as an open service in [24]. A schema of the methodology used to compute the rCBV_HAT_ is presented in Fig. 2. This methodology has been shown to obtain comparable rCBV values between centers using different clinical protocols, imaging protocols, or scanner models as reported in [17].

### Stratification of responsive patients

The stratification of responsive patients was made based on the *MGMT* methylation and tumor vascularity (i.e., rCBV_HAT_) markers. We generated two subpopulations, namely (1) the *MGMT* methylated, moderately vascularized tumors and (2) the *MGMT* unmethylated, highly vascularized tumors. Highly and moderately vascular glioblastomas were determined by a rCBV_HAT_ threshold (rCBVth) value. The rCBVth value was defined as the median rCBV_HAT_ of the study cohort.

### Statistical analyses

To analyze whether *MGMT* methylation was associated with differences in rCBV_HAT_ values, we compared the rCBV_HAT_ values for methylated and unmethylated *MGMT* populations using a non-parametric Mann-Whitney *U* test. In addition, we assessed the complementary prognostic information provided by *MGMT* methylation and rCBV_HAT_, by computing three different Cox proportional hazards regressions: (1) The first regression took into account only *MGMT* methylation status plus a set of relevant clinical variables (i.e., age at diagnostic, gender, and the extent of resection); (2) The second regression took into account only the rCBV_HAT_ value in the HAT habitat, plus the clinical variables; (3) The last regression was performed using both the *MGMT* methylation status and the rCBV_HAT_ value, plus the clinical variables. In order to take advantage of as many cases as possible for the Cox analysis, we used a mean imputation strategy [31] in cases that did not have information on the extent of resection. The goodness of fit of each of survival models was evaluated through the concordance statistic as defined by Harrell et al [32].

As suggested in the literature [6], we performed a Kaplan-Meier survival analysis to assess differences in OS between patient subpopulations. The log-rank test was performed to test for significant differences in OS. For censored cases, we set the date of censorship to the last date of contact with the patient or, in cases where this information was not available, the date of the last MRI exam.

Finally, to reduce biases in survival analyses, we analyzed whether the *IDH1* mutation was related to *MGMT* methylation by performing a Fisher test, and whether *IDH1*-mutated tumors have significantly different rCBV_HAT_ values performing a Mann-Whitney *U* test, using those cases where *IDH1* information was available.

### Software

The MRI data analysis was performed using ONCOhabitats [24], a freely available online service (www.onchabitats.upv.es). It provides a fully automated pipeline for the analysis of glioblastoma MRI studies including pre-processing, lesion segmentation, DSC perfusion quantification, and high angiogenic tumor delineation services, among others. Statistical analysis was performed with MATLAB (R2019a) and R (v3.6.0) [33].

## Results

### Characterization of patient cohorts

Of the 110 patients included from the NCT03439332 dataset, six exams (patients) were excluded because of processing errors with the automatic processing pipeline, five exams were excluded due to noise or MR artifacts that precluded DSC quantification (gamma-variate *R*^2^ goodness of fit < 0.95), one exam was excluded due to inability to differentiate between tumor vascularity and reactive meningeal enhancement, and two cases were excluded because MRI perfusion field of view did not cover the entire lesion (see Fig. 1). Of the remaining 96 patients, 43 (44.8%) presented the MGMT methylated. With a median follow-up of 391 days, median OS was 402 days; 15 patients were alive at the last data cut-off date and were censored in the survival analyses. Table 1 includes the most relevant demographic, molecular, and clinical features of the study cohort and summarizes the patients recruited. No significant relationships have been found between IDH1 mutation and MGMT methylation status (*p* = 0.39), nor between IDH1 mutation and rCBV_HAT_ values (*p* = 0.15).

**Table 1:** Summary of NCT03439332 study cohort

Initial no. of patients	110
Excluded no. of patients	14 (13%)
Included no. of patients	96 (87%)
Gender (f/m)	30/66
Age at diagnosis (years)	59.1 [32, 81]
Median survival (days)	402 [43, 1229]
*IDH1*	
-Mutated	5
-Wild type	58
-Unknown	33
*MGMT*	
-Methylated	43
-Unmethylated	53
Resection	
-Gross total	45
-Subtotal	41
-Biopsy	8
-Unknown	2
Location	
-Frontal	36
-Parietal	16
-Temporal	31
-Occipital	2
-Unknown	11

### Non-significant association between *MGMT* and rCBV_HAT_

Median rCBV_HAT_ in the whole population was 10.73. Median rCBV_HAT_ values in methylated and non-methylated *MGMT* subpopulations were 11.06 and 10.35, respectively. rCBV_HAT_ distributions did not differ significantly (Mann-Whitney *U* of 1062; *n* = 43 and *n* = 53, respectively, *p* > 0.05 two-tailed) in the methylated and the non-methylated subpopulations.

### *MGMT* methylation status and rCBV_HAT_ provide complementary prognostic information

In order to evaluate the association between rCBV_HAT_ (as a continuous variable) and *MGMT* methylation status with OS, we initially constructed three Cox proportional hazards (Cox-PH) regression models. The results of the three Cox-PH regression models obtained from NCT03439332 dataset are shown in Table 2. Results of the two first models including rCBV_HAT_ or MGMT status independently showed a significant association of *MGMT* status (HR: 2.01; 95% CI: 1.21–3.34; *p* = 0.007) with OS, but a non-significant association of rCBV_HAT_ (HR: 1.05; 95% CI: 0.99–1.11; *p* = 0.102) with OS. When evaluating both parameters in the multivariable Cox-PH model (Table 2), both were independently associated with OS: *MGMT* status (HR: 2.12; 95% CI: 1.27–3.52; *p* = 0.004) and rCBV_HAT_ (HR: 1.06; 95% CI: 1.00–1.12; *p* = 0.049). The performance of the model improved by increasing the concordance, decreasing the *p* values of each variable, and increasing the hazard ratios (HR), suggesting that *MGMT* methylation and rCBV_HAT_ provide complementary prognostic information.

**Table 2:** Results of the three Cox proportional hazards regression models obtained from NCT03439332 dataset

Clinical variable	Excluding rCBV_HAT_		Excluding MGMT meth.		All clinical variables	
	HR (95% CI)	*p* value	HR (95% CI)	*p* value	HR (95% CI)	*p* value
Age at diagnostic	1.00 [0.97, 1.02]	0.948	0.99 [0.97, 1.02]	0.629	0.97 [0.99, 1.02]	0.695
Gender	0.88 [0.51, 1.53]	0.653	1.03 [0.61, 1.75]	0.910	0.83 [0.48, 1.44]	0.512
Resection	1.79 [0.86, 3.73]	0.119	2.16 [1.02, 4.54]	0.043*	1.84 [0.87, 3.86]	0.109
*MGMT* meth.	2.01 [1.21, 3.34]	0.007*	-	-	2.12 [0.27, 3.52]	0.004*
rCBV_HAT_	-	-	1.05 [0.99, 1.11]	0.102	1.06 [1.00, 1.12]	0.049*
Concordance	0.59		0.59		0.61	

* Indicates significant difference (*p* < 0.05)

### rCBV_HAT_ improves the capability of *MGMT* to stratify responsive patients

We then evaluated the impact of *MGMT* methylation status in patients with highly and moderately vascularized tumors, as defined by the rCBV_HAT_ values. rCBVth, defined as the median rCBV_HAT_ of the study cohort, was established at rCBVth = 10.73.

We then analyzed differences in OS between patients with methylated and unmethylated *MGMT* promoters for the entire cohort, patients with highly vascularized tumors (rCBV_HAT_ ≥ 10.73), and patients with moderately vascularized tumors (rCBV_HAT_ < 10.73). In the cohort, 48 (50%) and 48 (50%) patients presented with highly and moderately vascularized tumors, respectively.

**Fig. 3:**
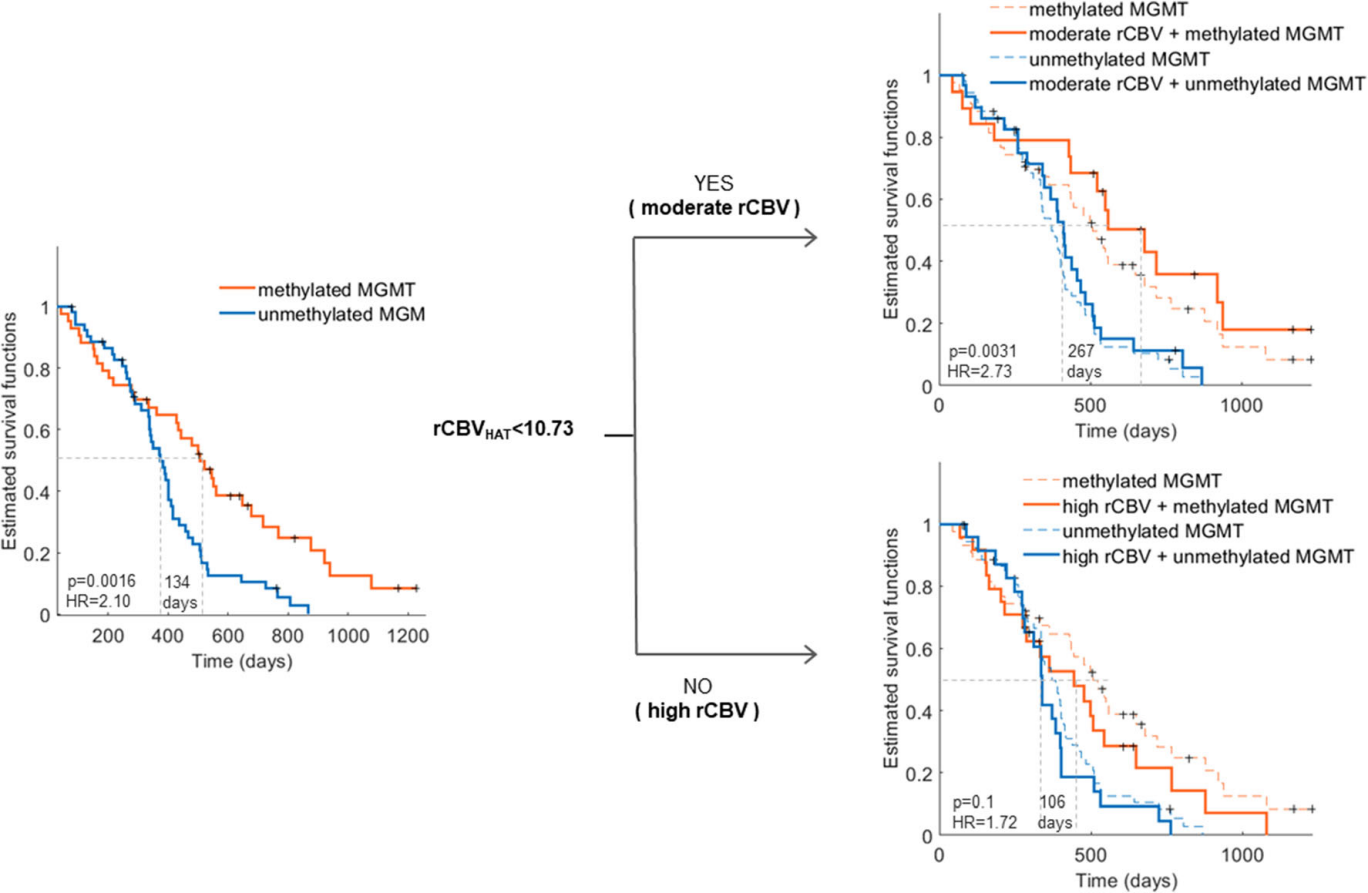
Kaplan-Meier curves showing the results of the stratification between moderate (rCBV_HAT_ < 10.73) and high (rCBV_HAT_ > 10.73) vascular subpopulations

We observed a significant association of *MGMT* methylation and OS (HR: 2.1 [95% CI: 1.33–3.33]; *p* = 0.002) (Table 3). In patients with moderately vascularized tumors (rCBV_HAT_ *<* 10.73), the association between *MGMT* methylation and OS was higher (HR: 2.73 [95% CI: 1.40–5.32]; *p* = 0.003) than in the general population. Finally, there was no association between *MGMT* methylation and OS in patients with high vascularity (rCBV_HAT_ ≥ 10.73) (HR: 1.72 [95% CI: 0.90–3.27]; *p* = 0.10). Kaplan-Meier curves for the four subpopulations are presented in Fig. 3.

**Table 3:** Results of the log-rank test of the Kaplan-Meier analysis for NCT03439332 cohort: (1) all, (2) moderate vascular (rCBV_HAT_ < rCBVth), and (3) high vascular (rCBV_HAT_ > rCBVth). For each subpopulation, the mean OS and number of patients with methylated MGMT and unmethylated MGMT are presented. Additionally, differences between OS (days), hazard ratios, area under the curve (AUC), and log-rank test resulting *p* value are presented

	No. of patients		Median OS					
	Meth. *MGMT*	Unmeth. *MGMT*	Meth. *MGMT*	Unmeth. *MGMT*	ΔOS	HR [95% CI]	*p*	AUC
All	43	53	507	373	134	2.10 [1.33–3.33]	0.0016	0.61
Moderate rCBV	19	29	678	411	267	2.73 [1.40–5.32]	0.0031	0.70
High rCBV	24	24	444	338	106	1.72 [0.90–3.27]	0.10	0.56

## Discussion

In this study, we present a potential role of tumor vascularity in the prognostic impact of *MGMT* methylation status in glioblastoma patients. As was expected from the literature, we observe a significant prognostic impact of *MGMT* methylation in glioblastoma patients treated with TMZ included in the NCT03439332 multicenter international dataset. Moreover, our results show that tumor vascularity may identify a subgroup of patients that exhibit a greater benefit from *MGMT* methylation. Once glioblastoma patients are dichotomized into highly and moderately vascularized tumor subgroups using rCBV, we observe a highly significant impact of *MGMT* status in patients with moderately vascularized tumors and only a non-significant trend in patients with highly vascularized tumors. From the comparison of the three multiparametric Cox survival analyses based on *MGMT* and rCBV_HAT_, we conclude that the information provided by both variables are relevant and complementary in predicting the prognosis of glioblastoma patients treated with TMZ. This implies that *MGMT* methylation assessment may provide incomplete information regarding prognosis, and that adding the characterization of tumor vascularization may improve estimation of responsiveness to TMZ. We propose a new classification of patients based on tumor vascularity, derived from the NCT03439332 multicenter international dataset. Our study proves the feasibility of using perfusion MRIs to identify subpopulations of glioblastoma patients with a higher likelihood of a beneficial effect of standard Stupp treatment (i.e., including TMZ), patients with methylated *MGMT*, and moderate vascularization in the contrast-enhancing tumor areas. On the contrary, patients with highly vascularized tumors will probably benefit less from TMZ, independently of the *MGMT* methylation status. We hypothesize that tumors with a lower vascularization may be potentially less aggressive, with a lower prevalence of molecular aberrations that may confer resistance to alkylating agents in the presence of a methylated *MGMT*.

Different studies have evaluated possible associations between *MGMT* methylation and tumor vascularity. A study by Hempel et al [34] (*n* = 100 including 24 glioblastomas) found significantly higher rCBV values in IDH wild-type glioblastomas with a methylated *MGMT* than in those with an unmethylated *MGMT*. Chahal et al [35] found higher levels of vascular endothelial growth factor receptor 1 (*VEGFR*-1) in unmethylated compared with methylated *MGMT* cells, hypothesizing that this should lead to an increased vascularization of the tumor. However, another small sample study (*n* = 24) by Moon et al [36] concluded that rCBV did not differ significantly between methylated and unmethylated *MGMT* tumors. In our study, based on a larger cohort, no significant relationship was observed between *MGMT* methylation and rCBV_HAT_. Due to the influence of *MGMT* on the effectiveness of therapies with antineoplastic drugs of alkylating agent class (i.e., TMZ), several studies suggest avoiding these therapies in those patients with non-methylated *MGMT* [6, 37]. The conclusions of this study further suggest that to obtain the most significant benefit, we should select among patients with methylated *MGMT* those with moderate vascularity.

The main limitation of the study is the lack of a complete molecular profile for all cases. Several studies already showed the importance of combining *MGMT* methylation status with other features when determining the patients who will benefit the most from it. Having methylated *MGMT* is an advantage especially when *TERT* expression is high [38] and progression-free survival is higher when both *MGMT* and mutant p53 expression are low [39]. Also, *MGMT* methylation displays better survival prediction performance when combined with *IDH1* mutations [40]. In this study, we have chosen to use CBV as the most relevant perfusion parameter for the analysis of the prognostic value of vascularity. However, in future work, it would be interesting to extend the study to include complementary markers derived from morphological [41], molecular [42], dynamic contrast-enhanced, and vessel architecture [43] imaging sequences that could help us to understand the complex vascular phenomena behind the results of this study.

This study demonstrates that combining *MGMT* methylation status together with the rCBV_HAT_ value obtained completely automatically from standard-of-care MRI studies through an online and open image analysis service (https://www.oncohabitats.upv.es) can provide a more reliable prognostic indicator for designing patient stratification strategies. This ensures the reproducibility of the obtained results and the immediate applicability of the proposed conclusions in the clinical trial setting. In conclusion, we consider that information of rCBV_HAT_ and *MGMT* methylation status should be included in randomization/stratification strategies of new clinical trials. Due to the joint implications of these two factors on patient survival, failure to consider these factors may lead to significant bias in the interpretation of the results from such studies.

## Electronic supplementary material

ESM 1 (DOCX 23 kb)

## Abbreviations

AUC: Area under the curve
DSC: Dynamic susceptibility contrast
FLAIR: Fluid-attenuated inversion recovery
HAT: Highly angiogenic tumor
HR: Hazard ratio
IDH: Isocitrate dehydrogenase
KPS: Karnofsky Performance Status
*MGMT*: *O(6)-Methylguanine-DNA methyltransferase*
MS-MLPA: Methylation-specific multiplex ligation-dependent probe amplification
MSP: Methylation-specific polymerase chain reaction
OS: Overall survival
rCBF: Relative cerebral blood flow
rCBV: Relative cerebral blood volume
RT: Radiotherapy
TMZ: Temozolomide
