# Supplementary material for: MGMT methylation may benefit overall survival in patients with moderately vascularized glioblastomas
Source: Eur Radiol. 2020 Oct 1;31(3):1738–47. doi: 10.1007/s00330-020-07297-4 (PMC7880975; doi:10.1007/s00330-020-07297-4)
Supplement: Supplementary file 1 — (DOCX 23 kb) [file 330_2020_7297_MOESM1_ESM.docx]

Supplementary Materials: Glioblastoma patients with a moderate vascular profile benefit the most from MGMT methylation

*Table SI.* *Summary of MRI acquisition parameters per hospital.*

|  |  | TR (ms) | TE (ms) | Matrix (mm) | Slice thickness  (mm) | FOV (cm^2^) | Number of dynamics |
| --- | --- | --- | --- | --- | --- | --- | --- |
| *HOSPITAL DE LA RIBERA* | T1 | 25 | 4.6 | 268x268 | 0.9 | 24x24 | - |
|  | T2 | 2000 | 120 | 320x199 | 5.0 | 23x18.3 | - |
|  | FLAIR | 1100 | 140 | 256x164 | 6.0 | 23x18.3 | - |
|  | DSC GE | 1650 | 40 | 116x116 | 2.2 | 24x24 | 80 |
| *HOSPITAL DE MANISES* | T1 | 500 | 20 | 304x241 | 5.0 | 24x24 | - |
|  | T2 | 2000 | 120 | 304x228 | 5.0 | 24x24 | - |
|  | FLAIR | 11000 | 140 | 256x209 | 6.0 | 24x24 | - |
|  | DSC GE | 836 | 30 | 128x128 | 5.0 | 24x24 | 40 |
| *CLINIC DE BARCELONA* | T1 | 12 | 4.68 | 256x256 | 1.0 | 24x24 | - |
|  | T2 | 3000 | 80 | 256x256 | 5.0 | 24x24 | - |
|  | FLAIR | 9000 | 164 | 256x256 | 5.0 | 24x24 | - |
|  | DSC GE | 1550 | 32 | 128x128 | 5.0 | 24x24 | 50 |
| *HOSPITAL VALL D’HEBRON* | T1 | 253 | 2.64 | 320x180 | 4.0 | 22x16.5 | - |
|  | T2 | 6100 | 91 | 512x326 | 4.0 | 22x17.5 | - |
|  | FLAIR | 9000 | 68 | 320x288 | 4.0 | 22x19.8 | - |
|  | DSC GE | 1450 | 45 | 128x128 | 5.0 | 23x23 | 60 |
| *AZIENDA OSPEDALIERO- DI PARMA* | T1 | 8.18 | 8.18 | 256x256 | 1.0 | 24x24 | - |
|  | T2 | 6500 | 65.90 | 160x160 | 4.0 | 24x24 | - |
|  | FLAIR | 12000 | 96.72 | 384x224 | 4.0 | 24x24 | - |
|  | DSC GE | 1500 | 30 | 128x128 | 4.0 | 24x24 | 60 |
| *CENTRE HOSPITALIER DE LIÈGE* | T1 | 13 | 4.76 | 256x218 | 1.0 | 25x25 | - |
|  | T2 | 5000 | 109 | 384x384 | 5.0 | 23x23 | - |
|  | FLAIR | 9000 | 90 | 256x173 | 5 | 23x23 | - |
|  | DSC GE | 1460 | 47 | 128x128 | 5 | 23x25 | 50 |
| *OSLO UNIVERSITY HOSPITAL* | T1 | 5.2 | 2.3 | 512x512 | 1.0 | 25.6x25.6 | - |
|  | T2 | 3800 | 84 | 896x896 | 3.0 | 22.0x22.0 | - |
|  | FLAIR | 4800 | 325 | 512x512 | 0.9 | 25.6x25.6 | - |
|  | DSC GE | 1500 | 25 | 128x128 | 5.0 | 25.6x25.6 | 100 |
